# Supplementary material for: Increasing prevalence of bacteriocin carriage in a six-year hospital cohort of E. faecium
Source: medRxiv. 2024 Jul 18:2024.07.17.24310592. Preprint. [Version 1] doi: 10.1101/2024.07.17.24310592 (PMC11275671; doi:10.1101/2024.07.17.24310592)
Supplement: Supplement 1 [file NIHPP2024.07.17.24310592v1-supplement-1.pdf]

medRxiv preprint doi: https://doi.org/10.1101/2024.07.17.24310592; this version posted July 18, 2024. The copyright holder for this preprint (which was not certified by peer review) is the author/funder, who has granted medRxiv a license to display the preprint in perpetuity.
This article is a US Government work. It is not subject to copyright under 17 USC 105 and is also made available for use under a CC0 license.

Supplemental data

Supplemental Table 1: Genes for enterocin A, bac43, and enterocin NKR-5-3B

Bacteriocin.	Gene	Length (bp)
Enterocin A<br>(Genbank accession number AF099088.1)	entA	198
	entI	312
	entF	147
	entK	1284
	entR	753
	entT	2154
	entD	1368
Bac43<br>(Genbank accession number AB178871.1)	bacA	225
	bacB	288
	mobC	381
	mobA	915
	repA	945
	repB	534
Enterocin NKR-5-3B<br>(Genbank accession number LC068607.1)	enkB	264
	enkB1	1140
	enkB2	519
	enkB3	591
	enkB4	498

Supplemental Table 2: PCR primers used for confirming bacteriocin gene presence

ID	Sequence	Description
bacA_fwd	TTGTCTAGCTGGCATCGGTA	Forward PCR primer targeting *bacA*
bacA_rev	GGACCATGATTAACCCAACC	Reverse PCR primer targeting *bacA*

medRxiv preprint doi: https://doi.org/10.1101/2024.07.17.24310592; this version posted July 18, 2024. The copyright holder for this preprint
(which was not certified by peer review) is the author/funder, who has granted medRxiv a license to display the preprint in perpetuity.
This article is a US Government work. It is not subject to copyright under 17 USC 105 and is also made available
for use under a CC0 license.

Supplemental Table 3: All bacteriocin cluster hits and the number of isolates they are found in.

**Bacteriocin cluster**	**Isolate presence**
Enterocin A cluster	2395
Bac43 full cluster	1406
Bac43 partial cluster	988
Enterocin NKR-5-3B cluster	466
Enterocin SE-K4 cluster	69
Enterocin P cluster	14
Enterocin B cluster	13
Bac32 cluster	12
Enterocin Xα cluster	10
Bac32 truncated cluster	5
Enterocin Xβ cluster	4
Enterocin L50b cluster	3
Enterocin L50a cluster	3
Enterocin Q cluster	2
Pneumolancidin cluster	1
Bac31 cluster	1
Enterocin P-like cluster	1
